# T cells in IgA nephropathy: role in pathogenesis, clinical significance and potential therapeutic target

**DOI:** 10.1007/s10157-018-1665-0

**Published:** 2018-11-07

**Authors:** Jakub Ruszkowski, Katarzyna A. Lisowska, Małgorzata Pindel, Zbigniew Heleniak, Alicja Dębska-Ślizień, Jacek M. Witkowski

**Affiliations:** 1 Department of Pathophysiology, Faculty of Medicine, Medical University of Gdańsk, Dębinki 7, 80-211 Gdańsk, Poland; 2 Department of Nephrology, Transplantology and Internal Medicine, Faculty of Medicine, Medical University of Gdańsk, Gdańsk, Poland

**Keywords:** Glomerulonephritis, IgA nephropathy, T lymphocytes

## Abstract

**Background:**

Immunoglobulin A nephropathy (IgAN), the most frequent cause of primary glomerulonephritis worldwide, is an autoimmune disease with complex pathogenesis. In this review, we focus on T cells and summarize knowledge about their involvement in pathophysiology and treatment of IgAN

**Methods:**

We reviewed the literature for (1) alterations of T cell subpopulations in IgAN, (2) experimental and clinical proofs for T cells’ participation in IgAN pathogenesis, (3) clinical correlations with T cell-associated alterations, and (4) influence of drugs used in IgAN therapy on T cell subpopulations.

**Results:**

We found that IgAN is characterized by higher proportions of circulatory Th2, Tfh, Th17, Th22 and γδ T cells, but lower Th1 and Treg cells. We discuss genetic and epigenetic makeup that may contribute to this immunological phenotype. We found that Th2, Th17 and Tfh-type interleukins contribute to elevated synthesis of galactose-deficient IgA1 (Gd-IgA1) and that the production of anti-Gd-IgA1 autoantibodies may be stimulated by Tfh cells. We described the roles of Th2, Th17, Th22 and Treg cells in the renal injury and summarized correlations between T cell-associated alterations and clinical features of IgAN (proteinuria, reduced GFR, hematuria). We detailed the impact of immunosuppressive drugs on T cell subpopulations and found that the majority of drugs have nonoptimal influence on T cells in IgAN patients.

**Conclusions:**

T cells play an important role in IgAN pathogenesis and are correlated with its clinical severity. Clinical trials with the drugs targeting the reported alterations of the T-cell compartment are highly desirable.

## Introduction

Immunoglobulin A nephropathy (IgAN) is characterized by the presence of immune complexes, predominantly containing polymeric IgA1, in the glomerular mesangium, which leads to glomerular injury [1]. It is the most common cause of primary glomerulonephritis in the world [1, 2]. However, the distribution of IgAN varies in different geographic regions; it is observed in up to 60% of all biopsies performed for glomerular disease in Asia compared with 30% in Europe and 10% in North America [3]. Geographical variability of detected IgAN prevalence can be explained by ethnic-based differences in the number of risk alleles as well as bias factors such as the presence of screening urinalysis and the differences in policies for performing renal biopsies [1]. IgAN can affect all ages, but is more common in children and young adults (20–30 years of age) [1]. Even though the disease usually follows a benign clinical course, it eventually results in end-stage renal disease (ESRD) in 15–20% of patients within 10 years and 30–40% of patients within 20–30 years after the first clinical presentation [1].

According to the well-accepted definition proposed by Suzuki et al., IgAN is an autoimmune disease with a multi-hit pathogenetic process. At least four processes (called “hits”) are necessary for the development of IgAN: (1) increased synthesis of circulating galactose-deficient-IgA1 (Gd-IgA1), (2) production of autoantibodies binding to Gd-IgA1, (3) formation of pathogenic Gd-IgA1-containing immune complexes, and then (4) mesangial deposition of these immune complexes resulting in mesangial cells activation and initiation of glomerular injury [4]. There are several factors involved in the etiology of IgAN. Recent reviews highlight the role of B cells and complement in the IgAN pathogenesis [5]. However, in this review, we focus on T cells and summarize knowledge about their involvement in IgAN pathogenesis, their clinical significance, and we also consider their role as a potential therapeutic target in the treatment.

## T cell subpopulations

T lymphocytes are a heterogeneous population of cells, characterized by the presence of a T-cell receptor (TCR)/CD3 complex on the cell surface, that participate in the adaptive immune response. The majority of human T cells have TCR composed of one α-chain and one β-chain, and so are called αβ T cells; while a relatively minor group of human T cells expresses a unique TCR composed of γ and δ chains (the γδ T cells). The αβ T cells are functionally subdivided into helper (Th), cytotoxic (Tc) and regulatory T (Treg) populations [6]. In contrast, γδ T cells are composed of two subsets that express either Vδ1 or Vδ2 gene; Vγ9Vδ2 T cells are the predominant subpopulation in human peripheral blood and will be called γδ T cells in this article.

Mature Th cells express the surface protein CD4 and can be differentiated into specific subtypes (Th1, Th2, Th9, Th17, Th22, Tfh). Each of the abovementioned subpopulations produces a specific set of cytokines essential for a successful response to infection [7].

Th1 and Th2 lymphocytes are the two main and best-known subpopulations of T helper cells. Th1 primarily participate in cell-mediated immunity and play an important role in the elimination of intracellular pathogens. They enhance cellular cytotoxicity and activate macrophages predominantly through production of interferon gamma (IFN-γ) [8]. In contrast, Th2 lymphocytes control humoral immunity, which is meditated by the immunoglobulins, and play an important role in the removal of multicellular parasites through production of interleukin (IL) 4, IL-5 and IL-13 [7]. Similarly to Th2, the Tfh are specialized in cooperation with B cells; they promote via IL-21 the survival and maturation of B cells, and such processes as immunoglobulin class switching and antibody affinity maturation [9, 10].

Th17 and Th22 lymphocytes are subpopulations defined by their ability to produce high concentrations of IL-17 and IL-22, respectively. Both subpopulations have a similar role: they take part in the immune response against extracellular bacteria, e.g., both stimulate epithelial cells to produce antibacterial peptides [11]. Additionally, Th17 lymphocytes secrete pro-inflammatory cytokines such as IL-17A and IL-17F, which act on a variety of cells upregulating the expression of pro-inflammatory cytokines, chemokines, and metalloproteases [11]. Hence, Th17 cells are considered to be involved in autoimmune processes. In contrast, IL-22 made by Th22 cells affects only epithelial cells of skin, digestive and respiratory tracts, and kidney [11, 12].

Tregs are the main population of lymphocytes characterized by high expression of FoxP3 transcription factor that counteract the excessive immune response, and protect the body from autoimmune responses. Treg can be divided into natural Treg (nTreg) arising in the thymus and inducible Treg (iTreg), which differentiate outside the thymus during the immune response. Another subdivision of the Tregs involves their functional state; thus resting and activated Tregs are described. Treg cells exert their suppressor effect on almost all cells in the immune system through secreted cytokines (mainly IL-10) and intercellular contact (through membrane-bound proteins such as CTLA-4) [7].

## Alterations of T cell subpopulations in IgA nephropathy

In Table 1, we summarized the findings concerning changes in frequency and function of Th1, Th2, Th17, Th22, Tfh, Tc, Treg and γδ T cells in patients suffering from IgAN. In short, IgAN is characterized by higher proportions of circulatory Th2, Tfh, Th17, Th22 and γδ T cells, but lower Th1 and Tregs (especially these induced and activated) [13–20]. Additionally, He et al. reported lower Th1/Th2 ratio among tonsillar lymphocytes of IgAN patients who suffered from tonsillitis compared to those with chronic tonsillitis without kidney disease [21], and Huang et al. observed a decreased frequency of tonsillar Tregs in IgAN patients [22].

**Table 1:** Changes in T cell subpopulations and serum cytokine concentrations in the peripheral blood of patients with IgA nephropathy

T cell subpopulation	Alterations compared with		References
	Healthy control	Other CKD as a control	
Th1			
% in PBL	↓/N	n.d.	[15, 16]
IFN-γ	↓/↑	n.d.	[15, 29, 78]
IL-2	↑	n.d.	[29, 78]
Th2			
% in PBL	↑	n.d.	[15]
IL-4	↑	n.d.	[29, 78]
IL-5	↑	n.d.	[15]
IL-6	↑	n.d	[13]
Th17			
% in PBL	↑	↑	[13–16]
IL-17A	↑	n.d.	[13, 15, 18, 29, 78]
Th22			
% in PBL	↑	↑	[14, 16]
IL-22	↑	↑	[14]
Tfh			
% in PBL	↑	n.d.	[17]
IL-21	↑	n.d.	[13, 17, 78]
Tc			
% in PBL	N	n.d	[62]
Treg			
Treg % in PBL	↓	n.d	[15, 19]
Activated Treg % in PBL	↓	n.d.	[13]
Resting Treg % in PBL	N	n.d.	[13]
iTreg % in PBL	↓	n.d.	[18]
nTreg % in PBL	N	n.d.	[18]
IL-10	↓/↑	n.d.	[13, 15, 18, 29, 78]
TGF-β1	↑/N/↓	n.d.	[13, 18, 32]
γδ T cells			
% in PBL	↑	n.d.	[20]

Disagreement in literature was shown using slash

*CKD* chronic kidney disease, *PBL* peripheral blood lymphocytes, ↑ increased versus control, ↓ decreased versus control, *N* unchanged versus control

Changes observed in the T cell subpopulations may be associated with the different genetic and epigenetic makeup of IgAN patients. Genetic studies confirm that there is Th1/Th2 imbalance in IgAN. Family-based study showed an association between IFN-γ polymorphism and higher susceptibility to the development of IgAN [23]. The + 874T/A polymorphism occurs in the binding site for transcription factor NF-κB (nuclear factor kappa-light-chain-enhancer of activated B cells), and the risk variant (+ 874A) is associated with decreased NF-κB binding affinity and decreased IFN-γ production in response to stimulation in vitro [23]. Thus IFN-γ, Th1-type cytokine, might have a protective role against the development of IgAN. Furthermore, genome-wide association studies (GWASs) have reported significant associations of IgAN development with polymorphisms of several genes involved in Th17 cells development and function [24]. One of the IgAN risk alleles is known for higher expression of *CARD9* gene. Protein encoded by this gene integrates signals stimulating Th17 differentiation following microbial exposition (mainly, but not limited to, fungal and mycobacterial) [24, 25]. Function of Th17 cells is strictly depended on their key transcription factor which can be degraded by the product of the *UBR5* gene. The expression of *UBR5* may be modified by another genetic polymorphism linked to increased risk of IgAN development [24]. Additionally, Th2- and Th17-polarization was associated with a deficiency of microRNA miR-155 in peripheral blood mononuclear cells (PBMC) of IgAN patients [15], which physiologically inhibits Th2 differentiation by suppression of IL-4 promoter transactivators: c-Maf and GATA3—the key transcription factors for Th2 cells [26].

Some studies suggest Th1 polarization but they are based on in vitro post-stimulation observations or animal models of IgAN [27, 28]. Meanwhile, human studies revealed either low [15] or only slightly elevated [29] IFN-γ serum concentrations in IgAN patients in contrast to clear significant elevation of Th2-type cytokines. It should be emphasized that IL-2, sometimes reported as a marker of Th1 polarization [27], is not restricted to Th1 subset; high amounts of IL-2 are also secreted by other Th subpopulations, activated Tc cells, NK T cells, and dendritic cells [30]. Furthermore, IL-2 is not secreted in all phases of Th1 development [8]. Strikingly, studies have shown that neither IL-2 production by PBMC nor serum IL-2 levels correlates with serum IgA levels, the severity of histologic changes in the kidneys of IgAN patients, or other clinical parameters [29, 31]. There are also a lot of controversies about the level of transforming growth factor β1 (TGF-β1) in patients with IgAN. A cohort study demonstrated elevated serum concentration of TGF-β1 in 100 Chinese patients, especially higher in advanced stages of IgAN [32]. It is supported by an observed deficiency of the miR-886 precursor that led to the overexpression of TGF-β [27]. However, another study, which included 63 Chinese patients, showed no significant difference in serum TGF-β1 level compared to the healthy control [13], and the smallest study had showed even a lowered serum level of TGF-β1 [18].

Studies agree on numerical deficiency and suggest a decreased immunosuppressive function of Tregs in IgAN [33]. Above-mentioned miR-155 deficiency might inhibit the maturation and differentiation of Treg cells of IgAN [15]. Ling-Wei et al. also reported elevated expression of miR-133a and miR-133b in PBMC of IgAN patients, and confirmed that these molecules inhibit Treg differentiation in IgAN through binding to FOXP3 mRNA with subsequent limitation of FOXP3 translation [19]. Next factor that might contribute to numerical deficiency of Tregs in IgAN is chronic tonsillitis; tonsillectomy for IgAN patients leads to some increase in the frequency of blood Treg cells, but still the observed numbers are lower than in healthy subjects [34]. On the other hand, the functional defect may be the result of IL-10 promoter polymorphisms associated with a reduced IL-10 production. These polymorphisms predispose to the development of IgAN in Korean and Chinese populations [35, 36], to faster progression in Caucasians [37], and even to recurrence of IgAN after transplantation [38]. However, some studies reported the contrary results [39]. Another factor affecting the function of Tregs in IgAN is an expression of CTLA-4; Jacob et al. reported that polymorphisms attributed to decreased *CTLA-4* gene expression were associated with higher proteinuria in IgAN patients [40]. However, the actual Treg’s CTLA-4 protein level was not investigated.

Finally, an excessive activity of immunoproteasomes may contribute to Th17/Treg disequilibrium in IgAN. It has been evidenced that immunoproteasome subunit coded by *PSMB8* gene is necessary for Th17 differentiation and inhibits Treg differentiation [41, 42]. It was demonstrated that PBMC of IgAN patients had higher expression of *PSMB8* gene [43, 44], especially those individuals with high proteinuria [43] or those experiencing greater annual loss of eGFR [45]. GWAS reported lower risk of developing IgAN by patients with polymorphism that lowers *PSMB8* gene expression [46].

## Elevated synthesis of Gd-IgA1—the role of Th2, Th17 and Tfh-type interleukins

The first hit in the multi-hit pathogenesis of IgAN is the appearance in the circulation of aberrantly glycosylated IgA1 with some hinge-region *O*-glycans deficient in galactose (Gd-IgA1) [4]. The origin of such autoantigens is still unclear; the most common hypothesis claims that it is a consequence of reduced galactosylation during post-translational modification of IgA1, but there are arguments that it might be the IgA1 produced by mucosally primed plasma cells [4, 5]. There are two possible mechanisms of reduced rate of galactosylation: (1) premature sialylation by ST6 N-acetylgalactosaminide alpha-2,6-sialyltransferase 2 (ST6GALNAC2), which prevents addition of galactose to N-acetylgalactosamine (GalNAc), and (2) lower activity of core 1 β1,3-galactosyltransferase (C1GALT1) due to its decreased expression or stability, the latter depends on the C1GALT1 specific chaperone 1 (C1GALT1C1), previously called COSMC [47]. In fact, Gd-IgA1-producing cells from IgAN patients have elevated expression of *ST6GALNAC2*, and decreased expression of *C1GALT1* and *C1GALT1C1* [47, 48].

**Fig. 1:**
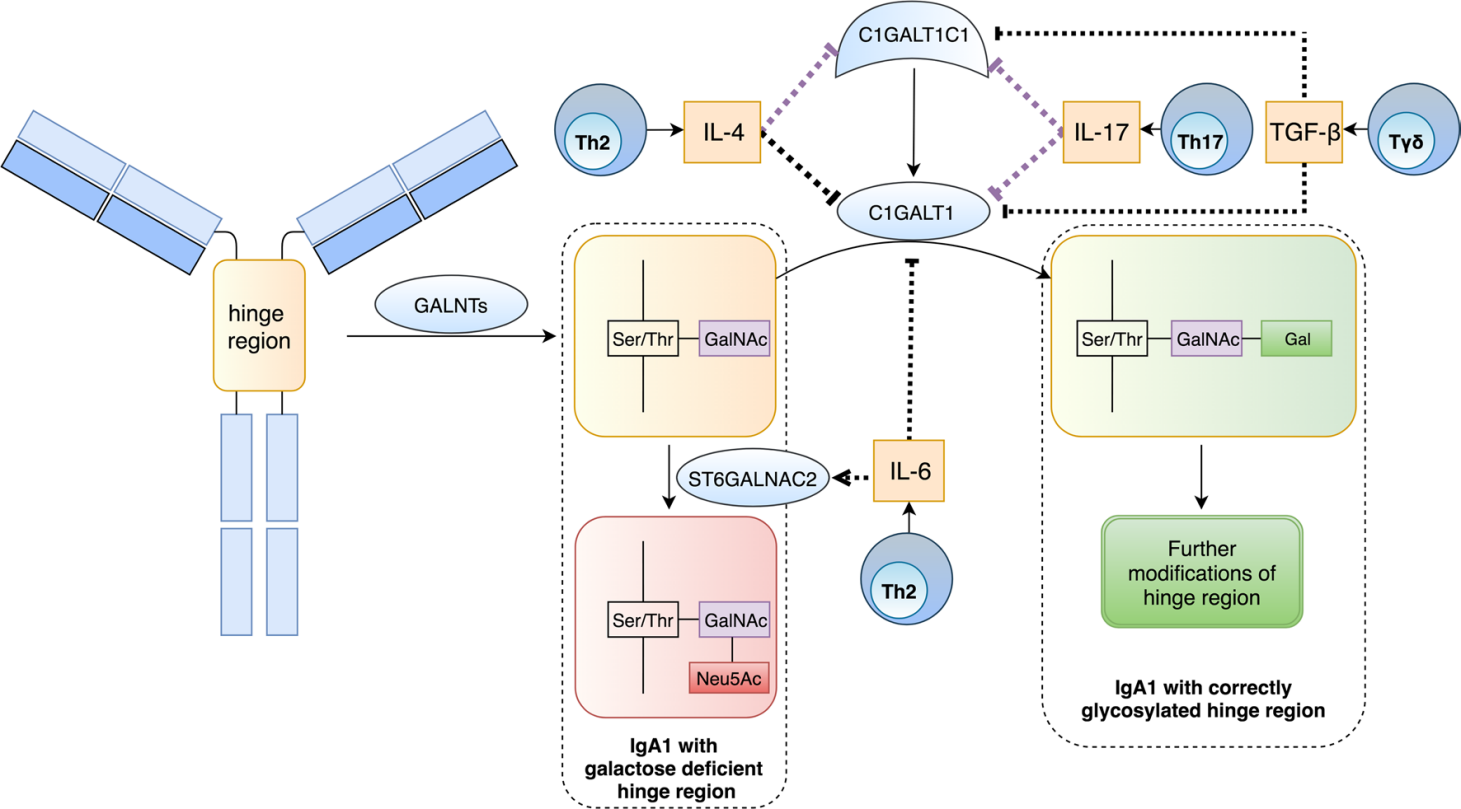
Involvement of T cells and their cytokines in posttranslational modification of IgA1 hinge region. The process starts with addition of *N*-acetylgalactosamine (GalNAc) to serine or threonine located in hinge region. Physiologically the process is continued by active C1GALT1, which adds galactose to GalNAc. Addition of sialic acid by ST6GALNAC2 prevents further galactosylation of GalNAc. In IgAN, IL-4 (Th2-type interleukin), IL-17 (Th17-type interleukin) and TGF-β are associated with decreased expression of C1GALT1 and its chaperon (C1GALT1C1). Additionally, IL-6 increases expression of ST6GALNAC2 and decreases expression of C1GALT1. All mentioned cytokines stimulate production of Gd-IgA1. Violet arrays—epigenetic mode of action; black arrays—unknown mode of action

**Fig. 2:**
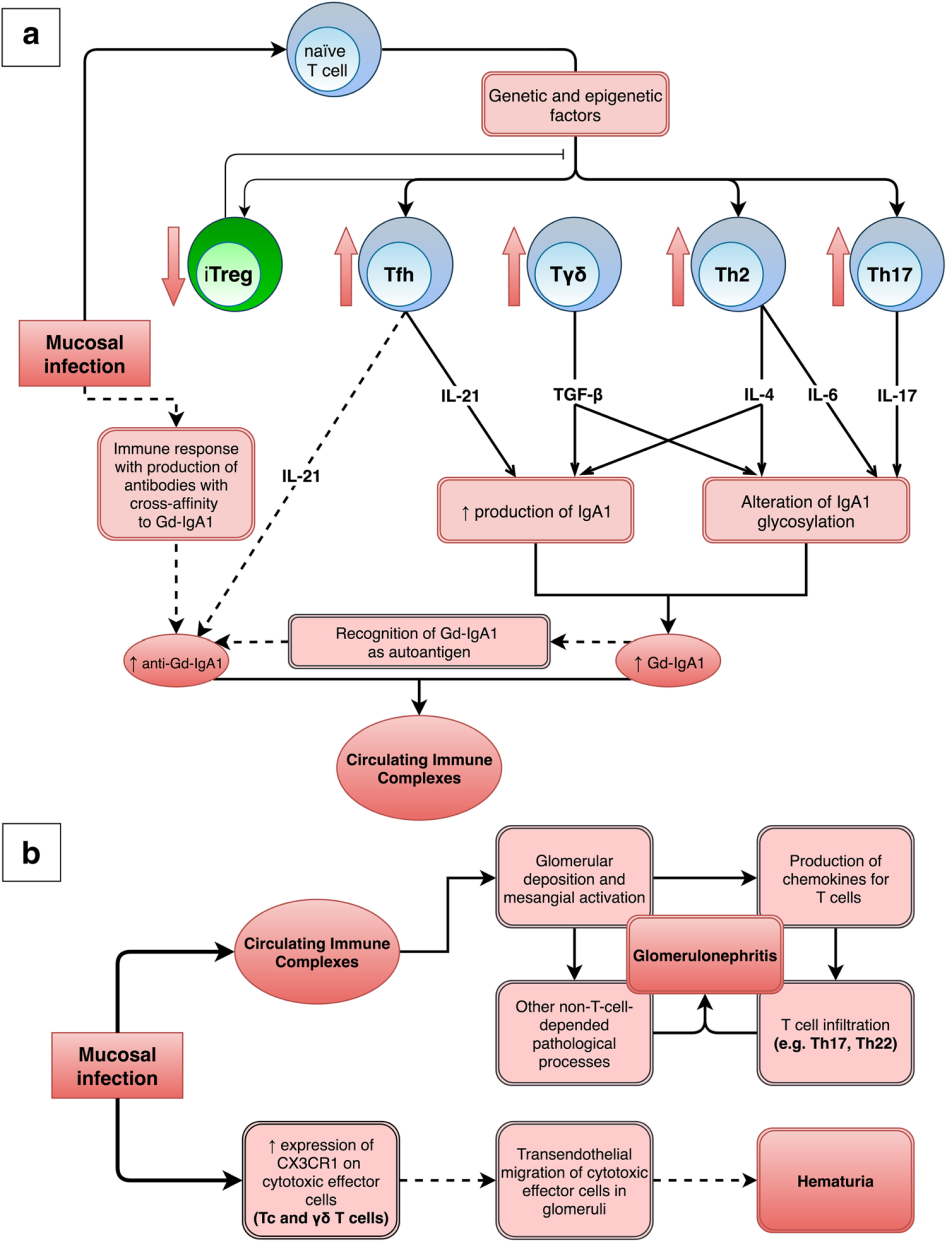
Involvement of T cells and their interleukins in the pathogenesis of IgAN. **a** Mucosal infection can stimulate the immune system to produce various cytokines, which beside participation in immune response against infection, may participate in the IgAN pathophysiology. IL-21 (Tfh-type interleukin) enhances IgA1 production and might participate in stimulation of anti-Gd-IgA1 production. IL-4 (Th2-type interleukin) and TGF-β enhance both IgA1 production and IgA1 glycosylation alteration. Both numerically and functionally deficient iTreg population cannot effectively suppress the defective immune response leading to formation of immune complexes. All these processes lead to the formation of circulating immune complexes. **b** Deposition of circulating immune complexes in glomeruli results in many pathological processes—such as T cells infiltration—that initiate and exacerbate the glomerulonephritis. Additionally, infection might result in hematuria through stimulation of transendothelial migration of cytotoxic effector cells (Tc and γδ T cells) from circulation to glomeruli. Solid and dashed lines represent confirmed and hypothetical links, respectively

Because mucosal infections coincide with IgAN exacerbation, it is believed that inflammation might impede IgA1 galactosylation in IgAN. Indeed, T cells might also participate in an aberrant IgA1 galactosylation process (Fig. 1). Already in 2008, Chintalacharuvu et al. confirmed that Th2-polarization promotes hypogalactosylation of IgA in a mouse model of IgAN [49]. Further studies on human B cell line and IgA1-secreting cells from IgAN patients revealed that IL-4 (but not IL-5) is responsible for this effect; it has been shown that IL-4 enhances IgA1 production and alters terminal glycosylation of secreted IgA1 (Fig. 2a) [50, 51]. The latter might be a result of down-regulation of C1GALT1 activity due to inhibition of both *C1GALT1* and its molecular chaperone *C1GALT1C1* expression [50, 51]. IL-4 promotes hypermethylation of CpG islands in *C1GALT1C1* gene promoter leading to the down-regulation of *C1GALT1C1* mRNA and related higher secretion of aberrantly glycosylated IgA1 from B cells [52]. What is more, B cells from IgAN patients seem to be more sensitive to IL-4 because the decrease in *C1GALT1C1* mRNA level induced by IL-4 was higher in IgAN B cells than in lymphocytes of both healthy children and children with other renal diseases [52]. IL-17 exhibits a similar mechanism of action; expression of *C1GALT1* and *C1GALT1C1* mRNA was significantly lower in B cell line stimulated by IL-17 [53]. Hypoglycosylation of IgA1 induced by IL-4 or IL-17 is reversed by 5-azacytidine [52, 53], proving that Th2- and Th17-derived interleukins disturb galactosylation of IgA1 through an epigenetic mechanism. Also IL-6, another pro-inflammatory cytokine, promotes hypogalactosylation of IgA1; stimulation of IgA1-secreting cells from IgAN patients with IL-6 increased ST6GALNAC2 activity and decreased activity of C1GALT1 through analogical changes in their genes expression [51].

The serum concentrations of both IgA and Gd-IgA1 are significantly higher for the IgAN patients compared with chronic kidney disease (CKD) patients or healthy people [54, 55]. Subpopulation of T cells—Tfh—might participate in elevated synthesis of IgA and Gd-IgA1 through IL-21 (Fig. 2a) [17]. This crucial interleukin upregulates in the mature B cells the expression of the activation-induced cytidine deaminase (AID), DNA-editing enzyme, which mediates IgA class switching during the differentiation of activated B cells into plasma cells [17, 56]. T-cell-dependent IgA class switching in B cells of IgAN patients can be stimulated also by TGF-β, cytokine produced by many cell types, e.g., γδ T cells and Tregs [20]. What is more important, TGF-β significantly decreases the mRNA levels of *C1GALT1* and *C1GALT1C1*, and thus contributes to the higher production of Gd-IgA1 (Fig. 1) [57]. However, TGF-β does not affect sialylation of IgA1 [57]. Meng et al. observed in IgAN patients very strong positive correlations between the serum concentration of TGF-β1 on one side and serum concentrations of total and secretory IgA and Gd-IgA1 on the other [32]. Toyabe et al. also reported a positive correlation between the proportion of γδ T cells and proportions of IgA-producing-B cells and serum IgA level in IgAN patients [20]. Less-specific T-cell-dependent mechanism of IgA switching is mediated via CD40L. This membrane-bound cytokine is present after activation on all investigated Th subsets (Th1, Th2, Tfh, Th17 cells), but not on Tregs [56, 58]. Besides the abovementioned T-cell-depended IgA class switching, the T-cell-independent manners of IgA switching may be involved in the intensive production of IgA and Gd-IgA1 in IgAN patients. This kind of IgA switching is mediated through molecules such as tumor necrosis factor ligand superfamily members 13 [59] and 13b [60, 61] (called April and BAFF, respectively) which are important for B cell development. Authors found out that serum levels of April and BAFF were increased in IgAN patients [59, 60]. Moreover, they have demonstrated that April induce an overproduction of Gd-IgA1 in cultured lymphocytes of IgAN patients [59].

However, some authors question the origin of circulatory Gd-IgA1 in IgAN; they argue that contrary to the common understanding, it is not the galactose-deficient IgA1 (problem in post-translation modification), but a misdirected normal mucosal form of IgA1 secreted into the circulation [5]. Pathological secretion of such IgA1 into the circulation is probably a result of defective trafficking during B cell maturation; plasma cells migrate into the bone marrow instead of settling down in the mucosa [5]. Batra et al. reported a similar homing pattern of Th cells; based on higher expression of integrin characterized for systemically homing cells (α4β1) rather than mucosal homing cells (α4β7) authors concluded that systemic homing Th cells may direct the aberrant systemic Gd-IgA1 production observed in IgAN [62]. Two places are frequently mentioned to be the site of lymphocyte activation in IgAN: tonsillar and gastrointestinal mucosa; the former is a reason of popularity of tonsillectomy in IgAN treatment. In accordance with the concept of T-cell-priming in tonsils, novel network meta-analysis showed that tonsillectomy (with steroids) was effective in inducing the remission of IgAN [63]. However, the same meta-analysis failed to show an efficacy of tonsillectomy (alone or with steroids) in either prevention of ESRD or doubling of serum creatinine levels. It might be a result of genetic and epigenetic make-up of entire T-cell population (not only in tonsils, but also in respiratory and intestinal mucosa). Therefore, tonsillectomy does not protect from both the activation of T cells in other mucosal compartments and the aggravation of IgAN. Indeed, high T cell infiltration in small intestine lamina propria was observed and strongly correlated with the serum IgA concentration in IgAN patients [64]. Additionally, a diminished repertoire of mucosal γδ T cells was observed in the guts of IgAN patients [65].

## Production of anti-Gd-IgA1 autoantibodies stimulated by Tfh

The seconnd hit in the IgAN pathogenesis is the production of autoantibodies against Gd-IgA1, predominantly in the IgG2 subclass [66]. Aberrantly glycosylated IgA1 might be recognized as an autoantigen, and thus the immune response against it may lead to the production of anti-IgA1-antibodies [67]. However, Novak et al. noticed that some pathogens, such as viruses and Gram-positive bacteria associated with upper respiratory infections, possess GalNAc-containing structures on their surfaces, which can mimic the GalNAc in the hinge region of Gd-IgA1 [67, 68]. Hence, such infections might stimulate the production of antibodies with cross-affinity to Gd-IgA1 which may lead to the formation of pathological immune complexes.

Further studies confirmed that anti-Gd-IgA1 autoantibodies did not originate from a rare germline variant, but from somatic hypermutation (SHM) of *VH* gene segments in anti-Gd-IgA1-producing cells [69]. IL-21 may promote SHM through increases of *AID* expression in the B cells of IgAN patients [17].

## Renal injury—Th2, Th17, Th22 and Treg cells participation in the last hit

Immunohistochemical staining of renal biopsies in IgAN indicates that kidneys are infiltrated mainly by αβ T cells, and additionally by γδ T cells in progressive IgAN [70, 71]. Multivariate analyses showed that tubulointerstitial T cell infiltration is independently associated with the progression of IgAN, which would suggest that T cells participate in renal injury in IgAN [72, 73].

Why do T cells infiltrate renal tissue? It turns out that Gd-IgA1 stimulates mesangium cells to produce chemokines for Th17 (CCL20) and Th22 cells (CCL20, CCL22 and CCL27): [74, 75]. Moreover, Th2 polarization might intensify the response of glomerular cells to IgA immune complexes and both directly and indirectly decrease glomerular filtration rate (GFR) [49]. Gan et al. confirmed higher renal expression of CCL20, CCL22, and CCL27 in IgAN patients; the elevation was even more pronounced in patients with accompanying tonsillitis [16]. Moreover, elevation of Th22 cells in patients’ peripheral blood was associated with worse histologic renal images; the percentage of Th22 cells was positively correlated with MEST scores. Authors concluded that tonsillitis aggravated renal injury in IgAN through induction of Th22 lymphocytosis, infiltration of renal tissue, and promotion of renal fibrosis by Th22 [16, 76].

On the other hand, using an animal model of IgAN, Huang et al. have evidenced that Tregs of IgAN patients cannot effectively suppress the deposition of IgA in the mesangial region, the expansion of the mesangial matrix, or the extensive proliferation of glomerular mesangial cells [33]. Additionally, the histological severity of the renal biopsy of IgAN patients tended to be worse in parallel with the decrease of blood Tregs frequency [34]. Therefore, it is hypothesized that the numerically and functionally defective Treg population cannot effectively suppress the renal injury in IgAN [34].

## Clinical correlations with T cell alterations

Majority of T cell subpopulations and interleukins alterations have been associated with clinical features of IgA nephropathy, such as the occurrence and severity of proteinuria, elevated serum creatinine concentration and reduced GFR, and hematuria (summarized in Table 2).

**Table 2:** Correlations between immunological and clinical features among IgAN patients

Clinical feature	Immunological feature	Coefficient of determination (*r*^2^)	References
Severity of 24-h proteinuria	Tonsillar Th1/Th2 ratio	0.6162	[21]
	miR-155 level in PBMC	0.5270	[15]
	Serum IL-21	0.4755	[17]
	Frequency of activated Tregs	0.3364	[13]
	Serum IL-17A	0.1225	[13]
Severity of 24-h albuminuria	sIL-2Ra level	0.0576	[77]
Estimated GFR	Frequency of activated Treg	0.4624	[13]
	Tfh cells	0.2824	[78]

The power of clinical severity determination is represented by coefficient of determination (*r*^2^)

The severity of 24-h proteinuria is positively correlated with serum IL-21 [17] and IL-17A [13]. Proteinuria-positive patients have a higher frequency of Th22 cells than proteinuria-negative IgAN patients and healthy people [14]. Moreover, higher proteinuria was observed in the patients possessing polymorphisms that caused a decreased expression of CTLA-4, immunosuppressive protein expressed on Tregs [40]. On the other hand, negative correlations were observed between the severity of 24-h proteinuria and tonsillar Th1/Th2 ratio [21], miR-155 level in PBMC [15], and frequency of activated Tregs [13]. Moreover, 24-h albuminuria is positively associated with sIL-2Ra level, a marker of continuous T cells activation [77].

Estimated GFR level is positively correlated with the frequency of activated Treg subset [13], and negatively with Tfh cells [78].

The density of Tc and Th cells in the renal interstitium is associated with the severity of erythrocyturia [79]. Cox et al. demonstrated that antigenic stimuli enhance CX3C chemokine receptor 1 (CX3CR1) expression on circulating blood cytotoxic effector cells (Tc and γδ T cells) of IgAN patients, which promotes glomerular transendothelial migration of lymphocytes and leads to a break in the continuity of the glomerular capillary wall, and subsequently to hematuria (Fig. 2b) [80]. CX3CR1-positive Tc cells are more frequent not only in the blood of IgAN patients with hematuria, but also in tonsils of IgAN patients [81]. Moreover, a synthetic analog of bacterial DNA upregulates the CX3CR1 expression on tonsillar Tc cells of IgAN patients [81]. Patients with significantly higher amount of glomerular and urinary fractalkine, the only ligand of CX3CR1, had recurrent episodes of gross hematuria [80]. Furthermore, disappearance of hematuria after tonsillectomy was associated with decrease in number of blood CX3CR1-positive Tc cells, while in patients with persistent hematuria the number of CX3CR1-positive Tc cells stayed unchanged [81].

Some of the biomarkers associated with T cells were analyzed in the context of renal outcome in the follow-up. As we mentioned, the degree of renal tubulointerstitial T cells infiltration is an independent prognostic biomarker of IgAN progressive course [72, 73]. Van Es et al. found in multiple regression analysis that intraepithelial Tc positive for natural killer cell granule protein 7 (expressed in activated T cells, in kidney, liver, lung and pancreas) was associated with the progression of IgAN in patients with normal or near-normal eGFR [82]. Additionally, high level (in the upper third tertile) of continuous T cell activation biomarker, sIL-2Ra, predicted IgAN progression to the combined end point, even after adjustment for the main clinical risk factors: time average albuminuria and GFR at baseline [77].

Clinical correlations and prognostic value of T cells’ biomarkers support the hypothesis that T cells, especially Tc, Th17, Th22 and Tfh, play a vital role in the pathogenesis and pathophysiology of IgAN.

## T cells as a therapeutic target of traditional and biological therapy

According to Kidney Disease: Improving Global Outcomes (KDIGO) Clinical Practice Guideline for Glomerulonephritis 2012, treatment of IgAN patients may include therapy with renin–angiotensin system (RAS) blockers, fish oil, corticosteroids, and non-steroidal immunosuppressive agents (cyclophosphamide, azathioprine and cyclosporine) [2]. One recently published study suggests that renoprotection (RAS blockers) is effective in preventing the progression of IgAN only if clinical and morphological risk factors are missing or modest [83]. It highlights the need for more aggressive treatment in patients with risk factors for the appearance of ESRD, such as proteinuria, hypertension, decreased eGFR, and severe histological lesions [84].

Considering a number of adverse effects, immunosuppression should be prescribed only for patients at the highest risk of developing ESRD [85]. Al-Lawati et al. suggested that immunosuppressive drugs in IgAN ought to modulate immune responses, including Gd-IgA1 production, glomerular and tubulointerstitial inflammation, and mesangial and endothelial cell proliferation [85]. All mentioned processes are dependent on the activity of T cells and their cytokines, which has been clearly demonstrated in our paper. What is more, even though B cells are more frequently described in the context of IgAN than T cells, recently published results of randomized, controlled trial of rituximab (anti-CD20 antibody) in IgAN, showed an inefficiency in the reduction of proteinuria, serum levels of Gd-IgA1 or antibodies against Gd-IgA1 [86]. This may encourage the nephrology community to focus on other targets: T cells and their interleukins.

### Corticosteroids

The most commonly used immunosuppression agents are corticosteroids. According to current guidelines, patients with GFR > 50 ml/min/1.73 m^2^ who fail to achieve levels of proteinuria below 1 g/day despite 3–6 months of optimized supportive care (including RAS blockers) are candidates for a 6-month course of systemic corticosteroid therapy [2]. The majority of recently published randomized controlled trials (RCTs) support the claim that corticosteroids reduce proteinuria and the probable progression of kidney function decline, but at the same time are associated with a number of adverse effects [87].

The knowledge about the precise effect of corticosteroids on T cells in IgAN is limited. Generally, corticosteroids are considered to inhibit production of both Th1- and Th2-type cytokines; probably with a more pronounced effect on Th1 cytokines in prolonged treatment [88]. In contrast, Zhang et al. observed elevation of IL-4 and IL-10 serum concentrations, Th2- and Treg-type cytokines, after 8–12 weeks of prednisone therapy in IgAN patients. Additionally, such corticosteroid treatment reduced the frequency of Tfh cells and the serum concentration of IL-21 [78].

To sum up, corticosteroids are an effective treatment for high risk patients with IgAN and the mechanism is at least partially T cell-dependent. However, given the elevation of IL-4, changes in T cell populations after treatment with corticosteroids seem to be not optimal for IgAN patients, thus more targeted therapeutics are needed. Admittedly, prednisone therapy can significantly reduce the levels of total IgA and Gd-IgA1 [89, 90] but, as Kosztyu et al. highlighted, levels of these immunoglobulins did not reach normal values [90].

### Azathioprine and cyclophosphamide

KDIGO guidelines suggest using corticosteroids combined with cyclophosphamide or azathioprine in IgAN patients only if there is crescentic IgAN with rapidly deteriorating kidney function [2]. However, the impact of these drugs on the course of IgAN is currently poorly documented. Studies on azathioprine in autoimmune diseases other than IgAN revealed reduction in γδ T [91] and Th17 cells but also an unfavorable reduction of Treg suppressive activity [92]. Reduction of number and suppressive activity of Treg was also reported for low dose cyclophosphamide; it seems that Tregs are more sensitive to cyclophosphamide compared with Th and Tc cells [93, 94]. On the contrary, administered intravenously high-dose cyclophosphamide (100–200 mg/kg divided over 2–4 consecutive days) is immunosuppressive through affecting all T cell subpopulations [95]. Unfortunately, clinical trials testing effectiveness of cyclophosphamide in IgAN treatment used low-dose protocols (about 1.5 mg/kg/day for 2–6 months) [2], which, according to current knowledge, might be responsible for its low effectiveness.

### Calcineurin inhibitors (CNIs): cyclosporine and tacrolimus

Recently published meta-analysis of seven RCTs indicates that the combination of CNIs and medium/low-dose corticosteroid is more effective in reducing proteinuria compared with the treatment with corticosteroid alone, suggesting a synergistic effect between CNIs and corticosteroids but without significant improvement in eGFR and higher incidents of gastrointestinal, neurological, and musculoskeletal symptoms [96].

Tacrolimus effectively inhibits key T cell activation pathways in T cells after kidney transplant [97]. A rat model of IgAN treated with tacrolimus revealed that usage of CNIs leads to an improvement of clinical features, along with reduction of serum concentration of TGF-β1, Th2-type cytokines (IL-4, IL-5), but elevation of Th1-type cytokine IFN-γ, which might have a protective role against the development of IgAN [98]. Median regression analysis of Gd-IgA1 serum changes in IgAN patients before transplantation and up to 6 months after transplantation revealed that the degree of exposure to the tacrolimus therapy correlated with a decrease of IgA1, but not Gd-IgA1 [89].

### Anti-thymocyte globulin (ATG)

Transplanted patients receive induction therapy just after transplantation to lower the risk of acute rejection during the early posttransplantation period. One of the commonly used and effective drugs is rabbit anti-thymocyte globulin (ATG) [99]. Studies have shown that ATG can effectively reduce the risk of recurrence of primary IgAN after renal transplantation [100, 101]. This observation will be verified after completion of an ongoing prospective, multicenter, randomized, open trial with a follow-up 5-year period called PIRAT (Prevention in Recipients With Primary IgA Nephropathy of Recurrence After Kidney Transplantation: ATG-F versus Basiliximab as Induction Immunosuppressive Treatment) [102].

The anti-inflammatory mechanism of ATG is based on induction of T cell apoptosis, while increasing the number of Treg cells and improving their function [103, 104]. The latter is attributed to elevated production of IL-4 and IL-13, Th2-type cytokines [105]. Also, renal recipients receiving ATG have prolonged depletion of Tfh cells [106].

### New targeted biological therapeutics

Advances in understanding IgAN pathobiology encourage us to target T cells and T cell cytokines in a more precise manner. Optimal immunotherapy in IgAN ought to reduce the activity of Th2, Tfh, Th17 and Th22 cells, while improving the function of Treg cells. All potential therapeutic targets with dedicated biologic drugs are summarized in Table 3. According to T cells’ participation in multi-hit pathogenetic process, clinical trials with the drugs included in Table 3, but currently untested in the IgAN context, would be desirable. Following reported correlations between T-cell interleukins and clinical severity of IgAN (summarized in Table 2), antibodies against IL-21 or inhibitors of IL-21 receptors should be especially tested.

**Table 3:** Proposed T-cell-dependent targets of biological therapeutics

Target	Mechanism of action	Drug
IL-4	Neutralization of IL-4	Pascolizumab
IL-4R/IL-13R	Antagonism of IL‑4/IL-13 receptor	Dupilumab, Pitakinra
IL-5	Neutralization of IL-5	Mepolizumab
IL-6	Neutralization of IL-6	Sirukumab
IL-6R	Antagonism of IL‑6 receptor	Tocilizumab
IL-12, IL-23	Inhibition of Th1 and Th17 differentiation through p40 inhibition	Ustekinumab
IL-17A	Neutralization of IL-17A	Secukinumab Ixekizumab
IL-21	Neutralization of IL-21	NNC0114-0006
IL-22	Neutralization of IL-22	Fezakinumab
TGF-β	Neutralization of TGF-β	Fresolimumab
TGF-βR	Antagonism of TGF-β receptor	Galunisertib

## Final conclusions

Excessive activity of T cells, especially the Th2, Tfh, Th17 and Th22 subpopulations, not only plays an important role in IgAN pathogenesis, but also is correlated with its clinical severity. The fact that 15–20% of patients within 10 years and 30–40% of patients within 20–30 years after the first clinical presentation progress to ESRD encourage us to a more aggressive treatment in patients with risk factors. However, the currently used immunosuppressive drugs for the treatment of IgAN are unspecific because they target all populations of T cells. In our opinion, optimal immunotherapy in IgAN should reduce the activity of specific subpopulations by modulation of cytokine levels or inhibition cytokine receptors, while simultaneously improving the function of Treg cells. Therefore, clinical trials with the drugs targeting the imbalance of the T cells compartment are highly desirable.
